# Accuracy of biomicroscopy, ultrasonography and spectral-domain OCT in detection of complete posterior vitreous detachment

**DOI:** 10.1186/s12886-023-03233-4

**Published:** 2023-11-28

**Authors:** Jasmin Zvorničanin, Edita Zvorničanin, Maja Popović

**Affiliations:** 1 Department of Ophthalmology, University Clinical Centre Tuzla, 75000 Tuzla, Bosnia and Herzegovina; 2 Faculty of Health Studies, University of Bihać, 77000 Bihać, Bosnia and Herzegovina; 3 Private Healthcare Institution “Vase Zdravlje”, 75000 Tuzla, Bosnia and Herzegovina; 4 Department of Medical Sciences, Cancer Epidemiology Unit, University of Turin and CPO-Piemonte, 10125 Turin, Italy

**Keywords:** Posterior vitreous detachment, Vitreoretinal interface, Macular hole, Epiretinal membrane, Optical coherence tomography, Biomicroscopy, Ultrasonography, Vitrectomy

## Abstract

**Background:**

To evaluate the accuracy of preoperative biomicroscopy (BM), ultrasonography (US), and spectral domain optical coherence tomography (SD-OCT) to determine complete posterior vitreous detachment (PVD) confirmed by intraoperative findings of triamcinolone acetonide-assisted pars plana vitrectomy (PPV).

**Methods:**

This prospective study included all consecutive patients admitted for surgical treatment of the epiretinal membrane (ERM) and macular hole (MH). The presence of complete PVD was determined one day before PPV using BM, US, SD-OCT. The preoperative findings were compared to the PVD status determined during PPV.

**Results:**

A total of 123 eyes from 123 patients were included in the study. Indications for PPV included ERM in 57 (46.3%), full thickness macular hole in 57 (46.3%) and lamellar macular hole in 9 (7.3%) patients. Complete PVD during PPV was observed in 18 (31.6%; 95%CI:18.7–49.9) patients with ERM and 13 (19.7%; 95%CI:10.4–33.7) patients with MH. The sensitivity of preoperative BM, US, SD-OCT was 48.4% (95%CI:30.2–66.9), 61.3% (95%CI:42.2–78.2) and 54.8% (95%CI:36.0–72.7) respectively. The specificity of preoperative BM, US, SD-OCT was 81.5% (95%CI:72.1–88.9), 90.2% (95%CI:82.2–95.4) and 85.9% (95%CI:77.0–92.3) respectively. With a prevalence of 25.2% of PVD in our sample the positive predictive value of preoperative BM, US, SD-OCT was 46.9% (95%CI:29.1–65.3), 67.9% (95%CI:47.6–84.1) and 56.7% (95%CI:37.4–74.5) respectively.

**Conclusion:**

Preoperative BM, US, and SD-OCT showed relatively low sensitivity but also good specificity in assessing complete PVD. A combination of all three diagnostic methods can provide a good assessment of the vitreoretinal interface state.

## Background

Posterior vitreous detachment (PVD) is defined as the separation of the posterior vitreous cortex from the internal limiting membrane of the retina [1, 2]. Assessment of PVD is important for presurgical planning and consultation in different ophthalmic conditions. The presence of complete PVD before retinal detachment (RD) surgery is associated with a higher primary anatomic success rate and may lead the surgeon to opt for pneumatic retinopexy as a possible treatment modality [3]. Furthermore, patients with RD and visible posterior hyaloid in the fellow eye are associated with a significantly higher risk of RD and need to be closely monitored at the time of PVD development [4]. During macular surgery, mechanical separation of the posterior hyaloid membrane is an important risk factor for retinal breaks [5]. Finally, a more favourable course and response to anti-VEGF therapy are associated with complete PVD in patients with age-related macular degeneration [6], retinal vein occlusion [7], and diabetic retinopathy [8].

The identification of the most efficient and reliable clinical technique for PVD detection still poses a significant challenge. Status of PVD can usually be assessed by slit-lamp biomicroscopy (BM), B-scan ultrasonography (US), and optical coherence tomography (OCT), with varying degree of accuracy [9–16]. Biomicroscopy could be limited by patients’ cooperation and examiner experience [9, 15]. Although considered as objective method for the PVD observation, due to its relatively low resolution, ultrasonography could be dependent on examiner ability to detect dynamic vitreous movements [9, 12, 15]. The use of OCT allows clinicians to delineate different retinal and choroidal pathologies, as well as to monitor therapeutic response to different treatment modalities [17–32]. Optical coherence tomography with its limited depth of imaging cannot visualize whole vitreous cavity and may provide high percent of false positive PVD findings [12]. To the best of our knowledge, there is only one previous study comparing results of all three diagnostic procedures with surgical findings in patients with different vitreomacular interface disorders [9].

Purpose of this study is to evaluate accuracy of pre-operative BM, US, and spectral domain OCT (SD-OCT) for determining complete PVD confirmed by intraoperative findings of triamcinolone acetonide-assisted pars plana vitrectomy (PPV).

## Methods

This was a prospective, consecutive, interventional case series based at the tertiary care Department of Ophthalmology, University Clinical Centre Tuzla, Bosnia and Herzegovina. The study included all patients admitted for surgical treatment of idiopathic epiretinal membrane (ERM) or macular hole (MH) in the period from January 1, 2015 to December 31, 2019. Exclusion criteria were the following: age less than 40, dense cataract or opaque ocular media that precluded fundus visualization, vitreous haze or bleeding, and all secondary causes in which the vitreous integrity could be jeopardized, including diabetes, high myopia, history of ocular trauma and previous vitrectomy [9, 11, 13]. The current study was approved by the University Clinical Centre Tuzla Ethics Committee (Approval number: 17/2301–6-14). Written informed consent was obtained from all patients after receiving an explanation of the investigative nature and intent of the study and tenets of the Helsinki Declaration were followed.

As part of preoperative preparation, all patients underwent a complete systemic evaluation that included general laboratory findings and a complete systemic examination to identify systemic diseases and drug use. To maximize the chance of achieving the appropriate vitreous evaluation, a complete preoperative ophthalmological evaluation (BM, US and SD-OCT) was performed the day before surgery [9, 14]. The first masked investigator performed slit-lamp BM with fully dilated pupils using a 78D lens (Volk Optical Inc., Enterprise Drive Mentor, OH, USA). A PVD was identified by the presence of a Weiss ring and/or a definitively detached visible posterior hyaloid membrane [9, 14, 15]. It was classified as the presence or absence of PVD in BM.

The second masked investigator performed ocular US using UD-800 (Tomey, Nagoya, Japan). Vertical and horizontal views were used and the mobility of the posterior vitreous was examined during saccadic eye movements with a high gain (90 dB), real-time, through-the-lid contact technique [13]. PVD status was considered when the posterior vitreous cortex was well defined and completely separated from the retina situated posterior to the equator and at the optic nerve head [9, 12, 13, 15]. It was classified as the presence or absence of PVD in US.

Spectral domain OCT images were obtained with fully dilated pupils using Cirrus HD-OCT (Carl Zeiss Meditec, Inc., Dublin, CA) with the Macular Cube 200 × 200 Combo protocol [33]. The protocol consisted of two perpendicular line scans centered at the fovea followed by a cube scan also centred at the fovea. The line scans were 6 mm in the transverse direction, had a 2 mm axial depth, and were composed of 1000 axial scans each. The cube scan was 6 × 6 mm, had an axial depth of 2 mm, and consisted of 200 × 200 axial scans [33]. We used the same methodology in accordance with the results of previous research that confirmed that partial and complete PVD can be accurately distinguished on OCT without including the optic nerve in the scan area as long as the imaging is centered and not shifted superiorly [34]. Therefore, SD-OCT scans were considered to have an acceptable position if the top of the scan was at least three “retina thicknesses” above the retinal pigment epithelium in the foveal center with an image quality of 8 and above [11, 34]. The thickness of the retina was measured at the nasal edge of the horizontal scan for each eye, unless this retina was pathologically thickened or thinned, in which case a temporal edge was selected. This criterion was selected to capture the anterior edge of the premacular bursa [11]. Both investigators independently interpreted the SD-OCT scans in a masked manner [12]. Posterior vitreous detachment was confirmed in SD-OCT when a hyperreflective linear signal was clearly separated from the neuroretina [12, 14]. Disagreement about PVD status was resolved by joint review of macular scans [11, 12]. It was classified as the presence or absence of PVD in OCT.

All results of preoperative examinations were compared with the findings of triamcinolone assisted 23 gauge 3-port pars plana vitrectomy (PPV) performed the following day. All surgeries were performed using Constellation (Alcon Laboratories, TX, USA) and recorded with a recording camera. After initial core vitrectomy, 4 mg/0.1 ml of triamcinolone acetonide suspension (40 mg/ml; Krka-Farma, Zagreb, Croatia) was injected and the posterior vitreous cortex evaluated. Vitreous was considered attached when firm vitreous attachment that had to be removed by vitreous cutter suction was observed during surgery. After complete vitrectomy, all patients underwent internal limiting membrane (ILM) peeling and the gas tamponade according to surgical indication [35]. In patients with ERM, the peeling of ERM was performed first, followed by staining and peeling of the ILM. The evaluation of PVD status was performed during the surgery and on the surgical video by both examiners [9, 14]. It was classified as the presence or absence of PVD during PPV.

Binary and categorical variables are reported as absolute numbers and percentages and were tested for differences between groups defined by the PPV-confirmed PVD status using the Chi-square test. Sensitivity, specificity, positive predictive value, and negative predictive value were calculated for the BM, US, and SD-OCT findings, using a PPV as a gold standard. Univariable and multivariable receiver operating characteristics (ROC) to obtain the values of the area under the curve (AUC) and the corresponding 95% confidence intervals (95% CI) were based on logistic regression models predicting the PPV-confirmed PVD result. Multivariable models were adjusted for sex and age. AUCs were compared using deLong test. All the analyses were performed using Stata version 15.1 (StataCorp, College Station, Texas, USA). The significance level for hypothesis testing was set at 0.05.

## Results

During the study period, a total of 129 patients underwent PPV for MH and ERM. A total of six patients were excluded from the study: 4 due to a history of ocular inflammation, and 2 with a history of trauma or surgery. Therefore, a total of 123 eyes from 123 patients were included in the study.

The average age of all included patients was 68.35 ± 0.69 (range 41—85) years. A total of 80 (65.04%; 95%CI:51.57–80.95) patients were female, with a female to male ratio of 1.86: 1. Indications for PPV included ERM in 57 (46.34%), full thickness macular hole (FTMH) in 57 (46.34%) and lamellar macular hole in 9 (7.32%) patients. Most of the patients were phakic 106 (86.18%; 95%CI:70.56–104.23), with a mean axial length of 23.12 ± 0.92 mm (range 20.7—25.94 mm). The average preoperative best corrected visual acuity on a decimal scale was 0.15 ± 0.11 (range 0.02–0.5). A total of 7 patients (5.69%; 95%CI:2.29–11.73) underwent surgery on their only functional eye. The mean preoperative intraocular pressure was 15.30 ± 2.20 (range 10–25) mmHg, and 7 patients (5.69%; 95%CI:2.29–11.73) have previously been diagnosed with glaucoma.

Complete PVD during PPV was observed in 31 (25.20%; 95%CI:17.12–35.77) patients (Table 1). Intraoperative PVD was found in 18 (31.58%; 95%CI:18.72–49.91) patients with ERM and 13 (19.7%; 95%CI:10.49–33.68) patients with FTMH (p = 0.096) (Table 2 and Table 3). All three diagnostic methods provided a different degree of accuracy regarding the vitreomacular interface status (Table 1).

**Table 1:** Overall sensitivity, specificity, positive predictive value, and negative predictive value with corresponding 95% confidence intervals (95% CI) of BM, US and SD-OCT for detecting complete PVD in different vitreo-macular interface conditions

	Total eyes	Intraoperative PVD (%)	Positive eyes (FP)	Negative eyes (FN)	Sensitivity (95% CI)	Specificity (95% CI)	Positive predictive value (95% CI)	Negative predictive value (95% CI)
Biomicroscopy	123	31 (25.2)	32 (17)	91 (16)	48.4 (30.2–66.9)	81.5 (72.1–88.9)	46.9 (29.1–65.3)	82.4 (73.0–89.6)
Ultrasonography	123	31 (25.2)	28 (9)	95 (12)	61.3 (42.2–78.2)	90.2 (82.2–95.4)	67.9 (47.6–84.1)	87.4 (79.0–93.3)
SD-OCT	123	31 (25.2)	30 (13)	93 (14)	54.8 (36.0–72.7)	85.9 (77.0–92.3)	56.7 (37.4–74.5)	84.9 (76.0–91.5)

*CI* Confidence Intervals, *FP* False positive, *FN* False negative, *PVD* Posterior vitreous detachment, *SD-OCT* Spectral domain optical coherence tomography

**Table 2:** Sensitivity, specificity, positive predictive value, and negative predictive value with corresponding 95% confidence intervals (95% CI) of BM, US and SD-OCT for detecting complete PVD in patients with ERM

	Total eyes	Intraoperative PVD (%)	Positive eyes (FP)	Negative eyes (FN)	Sensitivity (95% CI)	Specificity (95% CI)	Positive predictive value (95% CI)	Negative predictive value (95% CI)
Biomicroscopy	57	18 (31.6)	18 (9)	39 (9)	50.0 (26.0–74.0)	76.9 (60.7–88.9)	50.0 (26.0–74.0)	76.9 (60.7–88.9)
Ultrasonography	57	18 (31.6)	16 (3)	41 (5)	72.2 (46.5–90.3)	92.3 (79.1–98.4)	81.3 (54.4–96.0)	87.8 (73.8–95.9)
SD-OCT	57	18 (31.6)	17 (8)	40 (9)	50.0 (26.0–74.0)	79.5 (63.5–90.7)	52.9 (27.8–77.0)	77.5 (61.5–89.2)

*CI* Confidence Intervals, *FP* False positive, *FN* False negative, *PVD* Posterior vitreous detachment, *SD-OCT* Spectral domain optical coherence tomography

**Table 3:** Sensitivity, specificity, positive predictive value, and negative predictive value with corresponding 95% confidence intervals (95% CI) of BM, US and SD-OCT for detecting complete PVD in patients with FTMH and Lamellar macular hole

	Total eyes	Intraoperative PVD (%)	Positive eyes (FP)	Negative eyes (FN)	Sensitivity (95% CI)	Specificity (95% CI)	Positive predictive value (95% CI)	Negative predictive value (95% CI)
Biomicroscopy	66	13 (19.7)	14 (8)	52 (7)	46.2 (19.2–74.9)	84.9 (72.4–93.3)	42.9 (17.7–71.1)	86.5 (74.2–94.4)
Ultrasonography	66	13 (19.7)	12 (6)	54 (7)	46.2 (19.2–74.9)	88.7 (77.0–95.7)	50.0 (21.1–78.9)	87.0 (75.1–94.6)
SD-OCT	66	13 (19.7)	13 (5)	53 (5)	61.5 (31.6–86.1)	90.6 (79.3–96.9)	61.5 (31.6–86.1)	90.6 (79.3–96.9)

*CI* Confidence Intervals, *FP* False positive, *FN* False negative, *PVD* Posterior vitreous detachment, *SD-OCT* Spectral domain optical coherence tomography

The sensitivity of BM and SD-OCT was rather low for detecting complete PVD, both in patients with ERM and MH. SD-OCT seems to perform better in terms of sensitivity among patients with MH, but with very wide confidence intervals. Ultrasonography performed better compared to the other two diagnostic techniques in terms of sensitivity for all complete PVD (61.3; 95%CI:42.2–78.2), and this is particularly due to its diagnostic performance in detecting PVD in patients with ERM (72.2; 95%CI:46.5–90.3). All three diagnostic techniques have rather good diagnostic accuracy in terms of specificity. Ultrasonography was relatively accurate in diagnosing patients with PVD among those with ERM, as with an underlying prevalence of 31.6% of PVD, the probability of confirmed PVD among patients with positive US was 81.3% (95%CI:54.4–96.0).

**Fig. 1:**
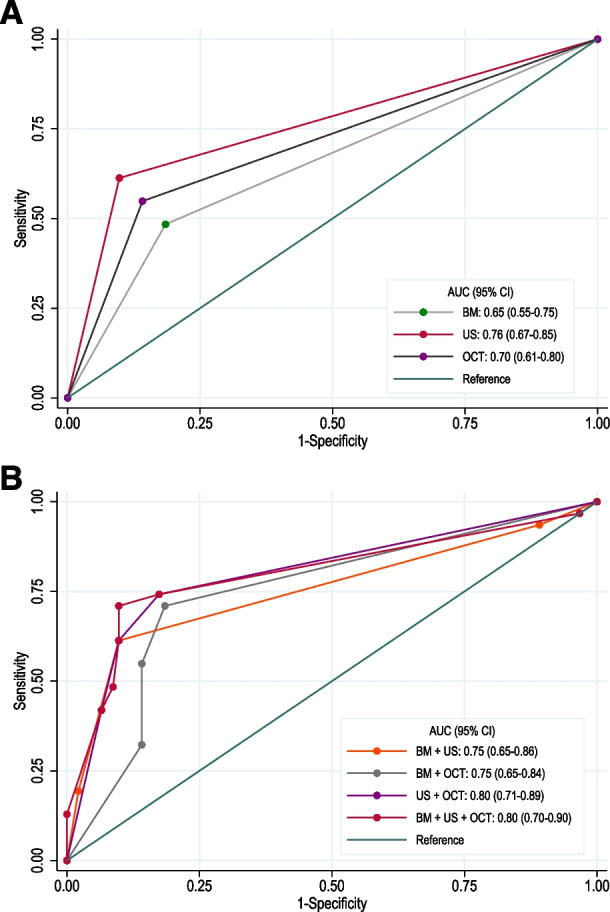
Receiver-operating-characteristic (ROC) curves of biomicroscopy (BM), spectral domain optical coherence tomography (OCT), and ultrasonography (US) alone (Panel A) and their combinations (Panel B) to distinguish patients with PVD from those without PVD as diagnosed during intraoperative PPV. Also indicated are the area under the ROC curve (AUC) and the 95% confidence interval (CI)

The combined diagnostic value of all three diagnostic methods is presented in Table 4. No differences were found in the prediction of PVD between BM alone and SD-OCT alone (adjusted model p = 0.13), and US alone and SD-OCT alone (adjusted model p = 0.66); US alone seems to perform better in predicting PVD compared to BM alone (p-value = 0.03 unadjusted, and p = 0.05 adjusted for sex and age) (Fig. 1. Panel A). The combination of US and SD-OCT increases the AUC to 0.80 (95%CI:0.71–0.89) and further to 0.89 (95%CI:0.83–0.95) when it comes to the age of the patient. The addition of BM did not improve the ability of the US + SD-OCT to distinguish PVD (p = 0.87 for unadjusted model and p = 0.89 for adjusted model). Female patients had a slightly higher prevalence of PVD than male patients (p = 0.057). Age was also strongly associated with the presence of PVD in the entire study population (p < 0.001). Therefore, including sex and age greatly improved the diagnostic ability of all the models (all AUCs above 0.80) (Fig. 1. Panel B).

**Table 4:** Unadjusted and adjusted ROC curve analysis predicting intraoperatively confirmed PVD

	**AUC (95% CI)**	
	**Unadjusted Models**	**Adjusted Models**^**1**^
BM	0.65 (0.55-0.75)	0.82 (0.74-0.90)
US	0.76 (0.67-0.85)	0.88 (0.81-0.95)
OCT	0.70 (0.61-0.80)	0.86 (0.80-0.93)
BM + US	0.75 (0.65-0.86)	0.88 (0.81-0.95)
BM+OCT	0.75 (0.65-0.84)	0.86 (0.80-0.93)
US+OCT	0.80 (0.71-0.89)	0.89 (0.83-0.95)
BM+US+OCT	0.80 (0.70-0.90)	0.89 (0.83-0.96)

*BM* biomicroscopy, *SD-OCT* spectral domain optical coherence tomography, *US* ultrasonography

^1^Adjusted for sex and age

## Discussion

In recent years, the evaluation of the posterior vitreous cortex status has become of great interest, while it can be decisive in assessing the best treatment modality for various retinal conditions [3, 6–9]. Only a few studies that have compared the preoperative reliability of diagnostic methods with intraoperative findings [9–11, 14, 16]. In this study, we have quantified the ability of BM, US, and SD-OCT to diagnose complete PVD compared to triamcinolone assisted intraoperative assessment in patients with different vitreoretinal interface disorders. To the best of our knowledge, this is the first prospective study focusing on the detection of complete PVD with all three methods compared to with intraoperative PPV in a relatively large sample of patients with ERM and FTMH.

The main finding of our study is that the three diagnostic procedures were unable to provide accurate information on PVD status compared to the intraoperative PPV findings. This result is consistent with the conclusions of earlier research [9, 11, 14, 16]. In this study, US was the most accurate non-invasive technique for assesing PVD before PPV, which confirms findings previously reported by Kičova et al. [9]. Although triamcinolone-assisted PPV is considered a gold standard for posterior vitreous visualization [16], it is also associated with a lower rate of reoperations [36]. Furthermore, considering the low sensitivity of preoperative diagnostic methods, the use of triamcinolone staining during PPV might be imperative to achieve adequate vitrectomy, resulting in the best possible postoperative anatomical and functional results [16].

The results of the current study indicate that complete PVD is less common, with only a quarter of all patients presenting this finding. Previous studies that analysed the reliability of different diagnostic modalities showed significantly different frequencies of PVD detected during PPV, ranging from 16 to 60% [5, 9, 11, 14]. It is important to note that these studies used different definitions of PVD staging, included different numbers of patients with various indications and preoperative characteristics. However, in patients with FTMH complete PVD is found in approximately 21% of patients [37], which is consistent with 19.7% presented in this study. On the other hand, in cases with ERM, the reported prevalence of intraoperative PVD is between 77.3% and 79.9% [38, 39], which is significantly higher than the 31.6% presented in this study. A possible explanation might be based on the concept of a complete and incomplete PVD [1], i.e. the criteria that were used in previous research. Histopathological studies confirmed that remnants of the vitreous cortex membrane frequently remain attached to the fovea after apparent complete PVD [40]. Unlike previous studies, all patients included in this study underwent complete vitrectomy with triamcinolone staining and ILM peeling. In this manner, we were able to detect and clean all vitreous remnants and reliably determine the state of PVD in all of our cases. Another explanation can be found in the selection of patients included in previous studies, while differences between studies in patient age, race, sex, or prevalence of systemic (diabetes) and ocular (myopia, pseudophakia) factors can certainly have an impact on PVD occurrence [41].

In this study BM has presented a sensitivity of 48.4% to determine the status of PVD by presence of the Weiss ring. The results of previous studies have shown significantly higher sensitivity of 76% [9] and 90% [14], in identifying complete PVD. Although BM can provide a dynamic, wide-angle observation of the posterior pole [14], it is also considered to be an investigator dependent method that is largely dependent on patient cooperation and transparency of the ocular media [9, 15]. In their study protocol, Stavrakas et al. [14] classified all dubious cases as non PVD during the BM examination. However, this protocol was not used in our study, and we have presented a high rate of false positive results and, consequently, a poor sensitivity. On the other hand, the presented specificity of 81.5% could be considered approximately equal to the results of previous research. This could imply, that in cases where the Weiss ring is not clearly visible during BM, it is more likely to be a partial PVD rather than a complete PVD.

Ultrasonography previously reported a sensitivity of 83% in the detection of complete PVD [8], which is better than 61.3% presented in the present study. This difference may be due to several factors. Interpretation of US findings could be subjective and dependent on the examiner’s ability to detect dynamic vitreous movements [9, 12, 15]. Furthermore, due to its relatively low resolution, even with higher gain and direct ocular contact, US has limitations regarding the detection of flat PVD [12]. This probably resulted in a high number of false negative findings and poor sensitivity in the present study, although we used dynamic ultrasonography in all of our cases. Nevertheless, the reported specificity of 90.2% could be considered within the scale of previous reports [9].

The application of OCT is useful for identifying various preretinal, retinal, subretinal, and choroidal changes. High-quality OCT images can serve as an excellent screening tool but also help to monitor response to different therapeutic modalities [17–32]. In the analysis of complete PVD, time domain OCT provided a correct assignment in only 12.5% of the cases and reported a high percentage (73.3%) of inadequate evaluations [9]. On the other hand, Hwang et al. used 6 mm SD-OCT images with visualization of vitreous approximately 900 µm above the retina in the foveal centre, and reported a sensitivity of 71% and a specificity of 88% [11]. This is better than our study, with a presented sensitivity of 54.8% and a specificity of 85.9%. Poor sensitivity in this study is consequent to a relatively high number of false positive cases, which may be the result of the shallower section of vitreous included in our SD-OCT images. However, our results are significantly better than those reported by Stavrakas et al. with a sensitivity of 37.5% and specificity of 31.3%, which also included only macular cube images [14]. Interestingly, three dimensional OCT images of the optic disc in a relatively small sample of patients provided the highest sensitivity of 97.4% with a reported specificity of 100% [10].

On the other hand, vitreoretinal adhesion or separation in the optic nerve can be inferred on a 6-mm macular OCT scan, although the optic nerve is not captured in the scan area [34]. Like previous research, we assumed that if the posterior vitreous cortex was visible on the top part of the scan, it was still attached to the optic nerve. On the contrary, we assumed that if the posterior vitreous cortex could not be visualized on the 6-mm OCT scan, it was separated from the optic nerve, that is, that a complete PVD was present. Therefore, 6-mm scans may be sufficient to assess PVD status, and visualization of the vitreoretinal interface at the optic nerve may be less important than previously claimed as long as the images are not shifted too far superiorly [34]. In this research, we insisted on high-quality images of the retina with the lowest possible position of the retinal image.

Optical coherence tomography imaging is an emerging standard of care in the setting of patients presenting with new flashes and floaters [42]. Enhanced depth imaging (EDI) for SD-OCT has further improved depth sensitivity, which enables detailed monitoring of choroidal structure and measurement of choroidal thickness [43]. Recent development of swept-source OCT (SS-OCT) has enabled imaging of larger areas with reduced motion artifact, and a better visualization of the choroidal vasculature [44]. The use of wide-field SS-OCT technology, which allows simultaneous observation of the optic nerve and macula, along with a greater depth of the vitreous included in the OCT image, will certainly enable a more reliable assessment of PVD state in the future [46]. More recent advances include a wide variety of new technologies, including wide-field colour photographs, quantitative fundus autofluorescence (qAF), adaptive optics (AO), and fluorescence lifetime imaging ophthalmoscopy (FLIO) [45]. However, it should be kept in mind that each technology has different capabilities and, to date there is no single modality capable of providing all the necessary information for a certain disease.

In this study, we have also analysed the combined value of all three diagnostic methods. The combined use of SD-OCT and US provided the best results, while the addition of BM did not provide a significant improvement. The rationale is simple, while SD-OCT has the ability to capture shallow PVD, US, on the other side can detect PVD that is highly detached over the retina and also visualize the optic nerve head. Furthermore, our findings indicate that the age and sex of patients, which are highly predictive of PVD status, when considered jointly in the models, significantly improve the diagnostic accuracy of the three diagnostic methods alone.

The strengths of this study include the prospective design and the availability of a wide range of preoperative and intraoperative details. We have insistently used only high-quality images and used the same preoperative and intraoperative protocol, including ILM peeling, in all our patients. The limitations of this study include the examiner related bias, with BM performed and interpreted by one examiner and US by another examiner, and SD-OCT and intraoperative findings analysed by both examiners. A possible source of error is a change in PVD status from examination to surgery [11, 16]. To minimize this possible bias, all patients included in this study underwent a complete standardized examination one day before the surgery [9, 14].

In conclusion, we present the comparative findings of three diagnostic procedures with the intraoperative findings of PPV. Preoperative BM, US, and SD-OCT are associated with relatively poor sensitivity and relatively good specificity for detecting complete PVD. A combination of all three diagnostic methods can provide a good insight into the condition of the vitreoretinal interface. A detailed preoperative examination and planning are necessary to achieve the best intraoperative and therefore postoperative results.

## Data Availability

The datasets generated and analysed during the current study are available in the Dryad repository, https://datadryad.org/stash/share/sbAj2hQrU8sfS9fO9e3qnCgijMbAYv9O1_x4U3bZC-I Citation: Zvorničanin, Jasmin; Zvorničanin, Edita; Popović, Maja (2022), Accuracy of biomicroscopy, ultrasonography and spectral-domain OCT in detection of complete posterior vitreous detachment, Dryad, Dataset, https://doi.org/10.5061/dryad.h44j0zpnp

## Abbreviations

AUCL: Area under the curve
BM: Biomicroscopy
CI: CONFIDENCE intervals
ERM: Epiretinal membrane
FN: False negative
FP: False positive
FTMH: Full thickness macular hole
ILM: Internal limiting membrane
MH: Macular hole
PPV: Pars plana vitrectomy
PVD: Posterior vitreous detachment
RD: Retinal detachment
ROC: Receiver operating characteristics
SD-OCT: Spectral domain optical coherence tomography
US: Ultrasonography
